# R on T Phenomenon Causing Ventricular Fibrillation during LV Angiography

**DOI:** 10.4103/1995-705X.76806

**Published:** 2010

**Authors:** Naveen Garg, Nagaraja Moorthy

**Affiliations:** Department of Cardiology, Sanjay Gandhi Postgraduate Institute of Medical Sciences, Lucknow-226 014, India

A 58-year-old diabetic female who underwent coronary angioplasty of left anterior descending coronary artery (LAD) using DES 1 year back was admitted with the diagnosis of acute coronary syndrome (NSTEMI). Repeat coronary angiography showed moderate instent restenosis in LAD stent and new onset severe disease in right coronary artery (RCA) and left circumflex coronary artery (LCX). LV angiogram was performed using pigtail catheter with 25 ml of non-ionic contrast injected at a rate of 15 ml/sec. During LV angiography, she developed multiple ventricular premature complexes(VPCs) which culminated into ventricular fibrillation. Immediately, the pigtail catheter was withdrawn and VF was reverted to sinus rhythm by DC cardioversion. Careful examination of recorded LV angiogram with simultaneous electrocardiographic recording revealed R-on-T phenomena (arrow in image) preceding the initiation of VF [Figure 1, Video 1]. A 12-lead electrocardiogram after successful defibrillation revealed a normal sinus rhythm with no ST-segment elevation. There was no neurological deficit following successful defibrillation. Later, she underwent successful CABG.

**Figure 1:**
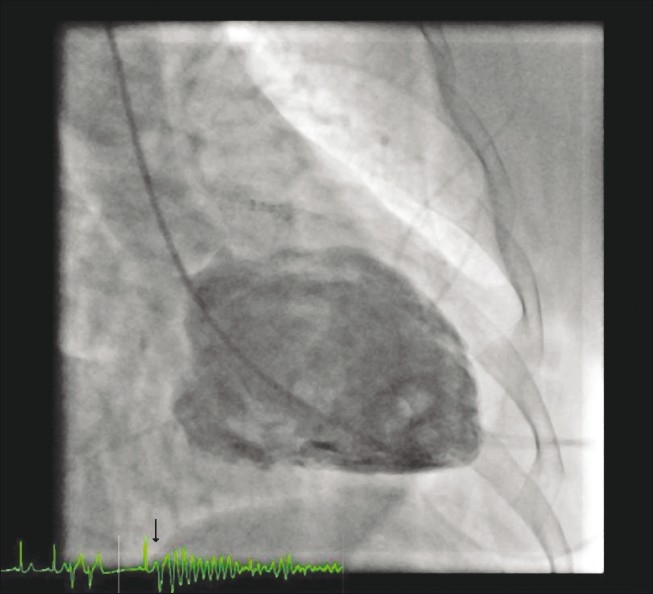
Simultaneous recording of electrocardiogram and LV angiography showing R-on-T phenomenon (arrow) causing ventricular fibrillation

## Video available at www.heartviews.org

Click here to view as Video 1

